# Impact of Troponin in Cardiomyopathy Development Caused by Mutations in Tropomyosin

**DOI:** 10.3390/ijms232415723

**Published:** 2022-12-11

**Authors:** Victoria V. Nefedova, Galina V. Kopylova, Daniil V. Shchepkin, Anastasia M. Kochurova, Olga I. Kechko, Vera A. Borzova, Natalia S. Ryabkova, Ivan A. Katrukha, Vladimir A. Mitkevich, Sergey Y. Bershitsky, Dmitrii I. Levitsky, Alexander M. Matyushenko

**Affiliations:** 1 Research Center of Biotechnology of the Russian Academy of Sciences, 119071 Moscow, Russia; 2 Institute of Immunology and Physiology of the Russian Academy of Sciences, 620049 Yekaterinburg, Russia; 3 Center for Precision Genome Editing and Genetic Technologies for Biomedicine, Engelhardt Institute of Molecular Biology, Russian Academy of Science, 119991 Moscow, Russia; 4 Department of Biochemistry, Faculty of Biology, Lomonosov Moscow State University, 119234 Moscow, Russia; 5 HyTest Ltd., 20520 Turku, Finland; 6 Engelhardt Institute of Molecular Biology, Russian Academy of Science, 119991 Moscow, Russia

**Keywords:** tropomyosin, troponin, muscle contraction, cardiomyopathy, isothermal titration calorimetry, thin filament, inherited cardiac disease

## Abstract

Tropomyosin (Tpm) mutations cause inherited cardiac diseases such as hypertrophic and dilated cardiomyopathies. We applied various approaches to investigate the role of cardiac troponin (Tn) and especially the troponin T (TnT) in the pathogenic effects of Tpm cardiomyopathy-associated mutations M8R, K15N, A277V, M281T, and I284V located in the overlap junction of neighboring Tpm dimers. Using co-sedimentation assay and viscosity measurements, we showed that TnT1 (fragment of TnT) stabilizes the overlap junction of Tpm WT and all Tpm mutants studied except Tpm M8R. However, isothermal titration calorimetry (ITC) indicated that TnT1 binds Tpm WT and all Tpm mutants similarly. By using ITC, we measured the direct K_D_ of the Tpm overlap region, N-end, and C-end binding to TnT1. The ITC data revealed that the Tpm C-end binds to TnT1 independently from the N-end, while N-end does not bind. Therefore, we suppose that Tpm M8R binds to TnT1 without forming the overlap junction. We also demonstrated the possible role of Tn isoform composition in the cardiomyopathy development caused by M8R mutation. TnT1 dose-dependently reduced the velocity of F-actin-Tpm filaments containing Tpm WT, Tpm A277V, and Tpm M281T mutants in an in vitro motility assay. All mutations impaired the calcium regulation of the actin–myosin interaction. The M281T and I284V mutations increased the calcium sensitivity, while the K15N and A277V mutations reduced it. The Tpm M8R, M281T, and I284V mutations under-inhibited the velocity at low calcium concentrations. Our results demonstrate that Tpm mutations likely implement their pathogenic effects through Tpm interaction with Tn, cardiac myosin, or other protein partners.

## 1. Introduction

Inherited cardiomyopathies are known to be caused by many missense mutations in different genes, including *TPM1*, which encode tropomyosin (Tpm) [1,2]. The muscle isoform of tropomyosin Tpm1.1 (or α-Tpm), in cooperation with other sarcomeric proteins (such as troponin complex), plays a critical role in the Ca^2+^-dependent regulation of striated muscle contraction. Mutations in the Tpm1.1 isoform are associated with two main types of cardiomyopathies: dilated (DCM) and hypertrophic (HCM). Despite the Tpm1.1 mutations being studied for a long time, many crucial questions are still waiting to be solved.

Tpm belongs to the coiled-coil protein superfamily and forms a prolonged interaction with the actin filaments along their length. The Tpm dimers polymerize in a head-to-tail manner through the interactions of their N- and C-termini [3]. The contractility of the myocardium is controlled by a Ca^2+^-dependent mechanism that recruits many proteins, including the troponin (Tn) complex [4,5]. In the absence of Ca^2+^, the complex of Tn with Tpm inhibits myosin binding to F-actin, while the Ca^2+^ binding to Tn during muscle activation forces Tpm to move away from the blocking position, thus allowing myosin interaction with actin and so muscle contraction [6]. The Tn complex is composed of three proteins named troponin T (TnT), troponin I (TnI), and troponin C (TnC) [7]. Among these proteins, TnI and TnT are presented by three isoforms: fast skeletal, slow skeletal, and cardiac isoforms, encoded by *TNNT3* and *TNNI2*, *TNNT1* and *TNNI1*, and *TNNT2* and *TNNI3* genes, respectively [8]. TnC represents two species: cardiac/slow skeletal and fast skeletal, encoded by two genes, *TNNC1* and *TNNC2*. TnT plays a key role in the anchoring of the Tn complex on thin filament via interactions with Tpm and also regulates the affinity of Tpm to actin filament [9,10,11].

According to previous works [12], TnT could be divided into two parts: TnT1 (residues 1–158 for rabbit fast skeletal troponin T, P02641) and TnT2 (residues 159–279), based on its proteolytic digestion site by chymotrypsin or CNBr. TnT contains two sites of interaction with Tpm. The first of them (site 1) is located in TnT1, and the other one (site 2) is in TnT2 [13]. As shown previously, the area of binding Tpm with the TnT site 1 includes the overlap junction of Tpm dimers, and site 2 interacts with the middle part of the Tpm molecule near Cys190 [13]. It was demonstrated that the TnT1 fragment decreases the ATPase activity of myosin [14] and the sliding velocity of Tpm-F-actin filaments in the in vitro motility assay [15]. Gollapudi et al. [16] have analyzed the functional significance of the N-terminal part of TnT containing site 1 on the activation of the thin filament and the kinetics of cross-bridge attachment in skinned myocardial preparations. Replacement of cardiac TnT site 1 with a skeletal one significantly reduced the Ca^2+^-sensitivity and the number of tightly attached cross-bridges and decreased the maximal force [16]. All this indicates that TnT site 1 is essential for myocardial contraction.

In a recent study, we focused on the five cardiomyopathy-causing mutations localized in the overlap junction of α-Tpm. The M8R, K15N, and A277V mutations are associated with DCM, and the M281T and I284V mutations relate to HCM. Hypertrophic cardiomyopathy is characterized by diastolic dysfunction and asymmetrical left ventricular hypertrophy. Its cellular hallmark is an increased sensitivity to Ca^2+^ [17]. The DCM phenotype in common is presented by an increased volume of the left ventricular chamber and systolic dysfunction [2].

Previously, it was shown that the M8R and K15N Tpm1.1 mutations located at the initial part of the Tpm N-termini dramatically decreased the affinity of Tpm to the actin filament [18,19,20]. Mutations M281T and I284V decreased the affinity of Tpm to F-actin, and A277V increased the affinity of Tpm to F-actin [20,21]. In addition, the A277V mutation stabilized the overlap junction [21,22], and the K15N mutation destabilized it [19,20]. The intrinsic stability of the Tpm mutants differs; Tpm A277V was more thermostable than Tpm WT and other mutants and formed more stable complexes with F-actin [18]. The K15N, M8R, and A277V mutations lowered the Ca^2+^-sensitivity [18,21,23].

Since the Tn complex is the main partner of Tpm in the regulation of cardiac muscle contractility, we investigated the interaction of these two proteins in more detail. It was shown previously that the critical disease-causing mutations of TnT also are located between 70 and 170 residues, i.e., in the zone corresponding to the TnT–Tpm binding region [24,25], indicating that interactions of TnT with Tpm play a crucial role in regulation of muscle contraction.

The goal of the present research was to investigate the role of cardiac Tn (particularly, its TnT1 fragment) in the development of cardiomyopathies caused by Tpm mutations in the overlap junction.

## 2. Results

### 2.1. Effect of TnT1 Fragment on the Binding Affinity of Tpm Mutants to F-Actin

**Figure 1:**
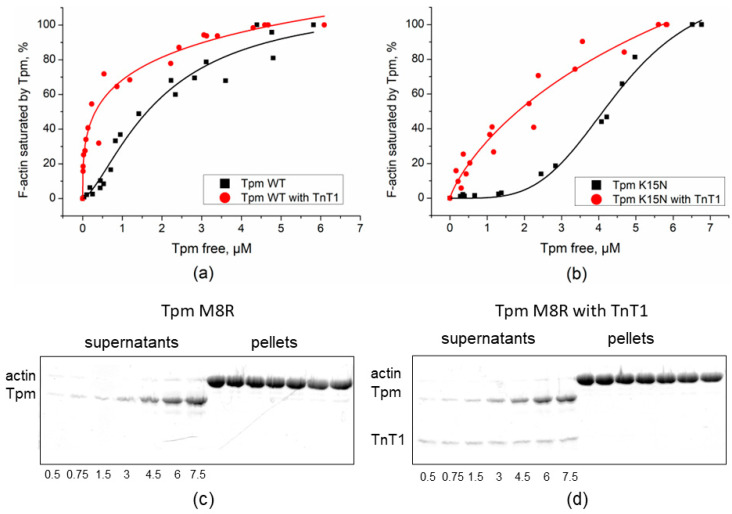
The affinity of the Tpm WT and N-terminal mutants Tpm M8R and K15N mutants to F-actin in the presence of TnT1. Results obtained by co-sedimentation assay are plotted as a fraction of F-actin saturated by Tpm against the concentration of free Tpm found in the supernatant. (**a**,**b**) The affinity of the Tpm WT and Tpm K15N. The assay was performed in buffer containing 200 mM NaCl. The estimated K_50%_ values were 1.61 ± 0.24 µM for Tpm WT alone and 0.3 ± 0.16 µM for Tpm WT in the presence of TnT1 (**a**) and 4.1 ± 0.01 µM for Tpm K15N alone and 1.8 ± 0.6 µM for Tpm K15N in presence of TnT1 (**b**). (**c**,**d**) SDS-PAGE of co-sedimentation assay of Tpm M8R alone (**c**) and in the presence of TnT1 (**d**) with F-actin. Total concentrations (µM) of Tpm in the samples are listed under gels.

**Figure 2:**
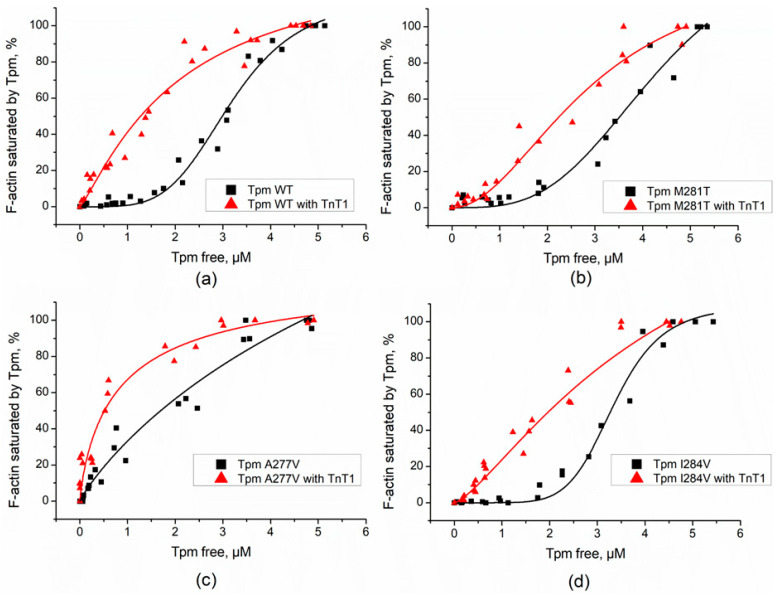
The affinity of the Tpm WT and the C-terminal mutants to F-actin in the presence of TnT1. Results obtained by the co-sedimentation assay are plotted as a fraction of F-actin saturated by Tpm against the concentration of free Tpm found in the supernatant. The estimated K_50%_ were (**a**) 3 ± 0.2 µM for WT alone and 1.25 ± 0.11 µM for Tpm WT with TnT1, (**b**) 3.5 ± 0.22 µM for M281T alone and 2.2 ± 0.18 µM for M281T in presence of TnT1, (**c**) 1.4 ± 0.21 µM for A277V alone and 0.5 ± 0.01 µM in presence of TnT1, and (**d**) 5.27 ± 0.25 µM for I284V alone and 1.96 ± 0.23 µM in presence of TnT1.

The Tpm mutants’ affinity to F-actin has been established in previous works [20,21]. As known, TnT increases Tpm affinity to F-actin [24]. To investigate the influence of the TnT1 fragment on the binding of cardiomyopathy-causing Tpm mutants to F-actin, we applied a co-sedimentation assay. The samples containing F-actin and Tpm mutants either with or without TnT1 fragment were compared by the K_50%_ parameter corresponding to the Tpm concentration at which half of the actin became saturated (Figure 1 and Figure 2). Head-to-tail interactions of Tpm are known to be reduced at higher ionic strength. For this reason, we used different ionic strengths: 200 mM NaCl-containing solution for M8R and K15N mutants and 255 mM NaCl-containing solution for A277V, M281T, and I284V mutants.

As expected, TnT1 increased the affinity of Tpm WT to F-actin almost five-fold (Figure 1a). The K15N mutation, which is known to decrease affinity of Tpm to actin, significantly restored this property in the presence of TnT1 fragment (Figure 1b). The K_50%_ value changed from 4.1 ± 0.01 µM for K15N Tpm alone to 1.8 ± 0.6 µM in presence of TnT1. The M8R mutant did not bind F-actin either with or without TnT1 (Figure 1c,d). All C-terminal mutants with TnT1 had a higher affinity to F-actin than N-terminal mutants (Figure 2). TnT1 increased the Tpm–F-actin affinity of all C-terminal mutants. The K_50%_ value of A277V Tpm with TnT1 was even higher than that of WT Tpm (see Figure 2a,c).

### 2.2. Effect of the TnT1 Fragment on the Thermally Induced Dissociation of Tpm–F-Actin Complexes

**Figure 3:**
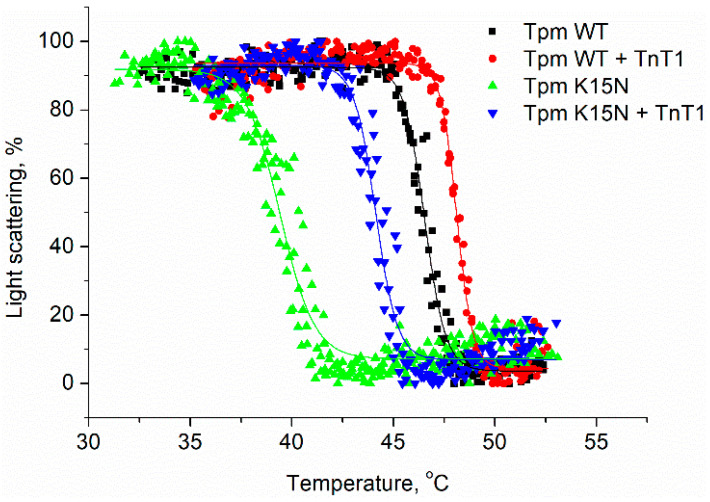
Normalized temperature dependencies of dissociation of the complexes of F-actin with the Tpm WT or Tpm K15N mutant in the absence or presence of TnT1. A 100% value corresponds to the difference between the light scattering of Tpm–F-actin complexes measured at 25 °C and that of pure F-actin stabilized by phalloidin, which was temperature-independent within the temperature range used. T_diss_ values for WT Tpm and Tpm mutants are presented in Table 1.

To examine whether the TnT1 could affect the stability of Tpm complexes with F-actin, we applied the thermally-induced dissociation assay. Light scattering of the Tpm–F-actin complex reduces with a rise in temperature and reaches the level of light scattering of bare F-actin at complete Tpm dissociation from actin. The addition of TnT1 increased the stability of Tpm–F-actin complexes both for Tpm WT and cardiomyopathy-causing Tpm mutants (Table 1). For most of the mutants, TnT1 increased T_diss_ by 1.4–1.8 °C (Figure 3 and Table 1). For the K15N mutant, which had the weakest overlap junction between Tpm dimers, in line with previous work [22], the T_diss_ of the complex increased by about 5 °C.

According to our data TnT1 does not change the light scattering of F-actin; therefore, it was natural to assume that its effect on the thermal stability of the Tpm–F-actin complex is implemented via strengthening of the Tpm strand on the F-actin surface.

### 2.3. Interaction of TnT1 Fragment with Tpm Overlap Junction

**Figure 4:**
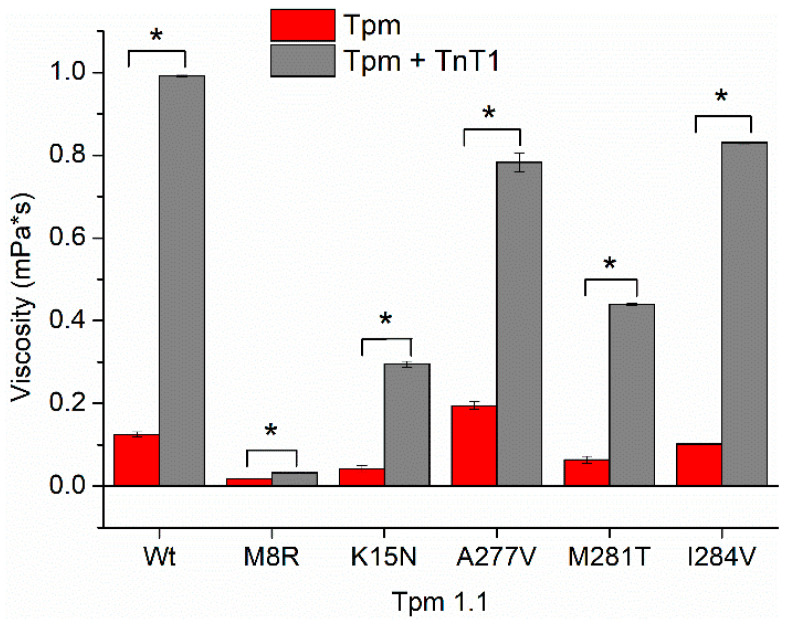
Excess viscosities of Tpm mutant solutions in the presence or absence of cardiac troponin T fragment (TnT1) over buffer viscosity. * Statistical differences in the excess viscosity of Tpm and Tpm + TnT1, *p* < 0.05.

To investigate the effect of TnT1 on the stability of the overlap junction, we measured the excess viscosity of solutions containing Tpm WT or Tpm mutants, both with and without TnT1 (Figure 4 and Table 2). The binding of TnT1 to Tpm WT resulted in an eight-fold increase in solution viscosity indicating an improvement in the formation of the overlap junction. TnT1 also increased the excess viscosities of Tpm with C-terminal mutations. Consistent with previous studies, the highest viscosity was shown for the A277V Tpm mutant [21,22]; however, TnT1 increased the viscosity of this mutant to a lesser extent compared to Tpm WT.

TnT1 only slightly increased the solution viscosity of the M8R Tpm mutant, known to strongly disrupt the end-to-end interactions [20]. In contrast, TnT1 recovered the end-to-end interaction, which was significantly suppressed by the K15N mutant. Note that in the presence of TnT1, none of the Tpm mutants achieved the viscosity of Tpm WT (Figure 4).

### 2.4. Effect of TnT1 on Actin–Myosin Interaction

**Figure 5:**
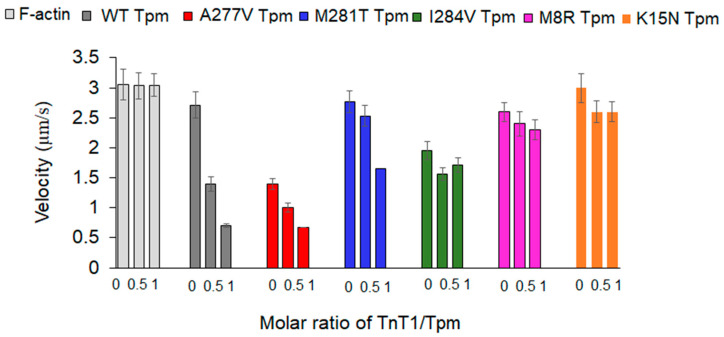
Effect of TnT1 on the sliding velocity of the F-actin-Tpm filaments, containing Tpm with cardiomyopathy mutations, over myosin in the in vitro motility assay.

To study the effect of TnT1 on the actin–myosin interaction, we analyzed the dependence of the sliding velocity of F-actin-Tpm filaments on the TnT1 concentration in the in vitro motility assay. We found that TnT1 does not affect the velocity of bare F-actin but decelerates the movement of thin filaments containing Tpm WT in a dose-dependent manner (Figure 5). TnT1 decreased the velocity of the filaments with Tpm A277V and Tpm M281T to a lesser degree than those with Tpm WT. As in the case of bare F-actin, TnT1 did not affect the velocity of filaments containing Tpm M8R, Tpm K15N, and I284V.

### 2.5. Effects of cardiomyopathy Tpm Mutations on the Ca^2+^-Regulation of the Actin–Myosin Interaction

**Figure 6:**
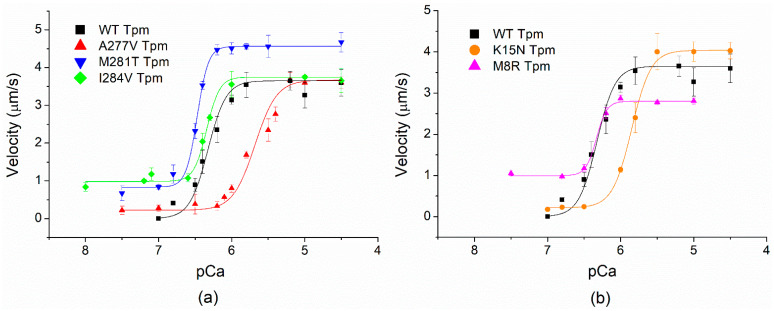
Effects of Tpm cardiomyopathy-causing mutants on Ca^2+^-dependent sliding velocity of regulated thin filaments. (**a**) Effects of the C-terminal mutants A277V, M281T, and I284V; (**b**) effects of the N-terminal mutants M8R and K15N. Each data point is the mean ± SD from three experiments. The data were fitted to the Hill equation. The pCa_50_ values at which the sliding velocity was half-maximal are shown in Table 3.

**Figure 7:**
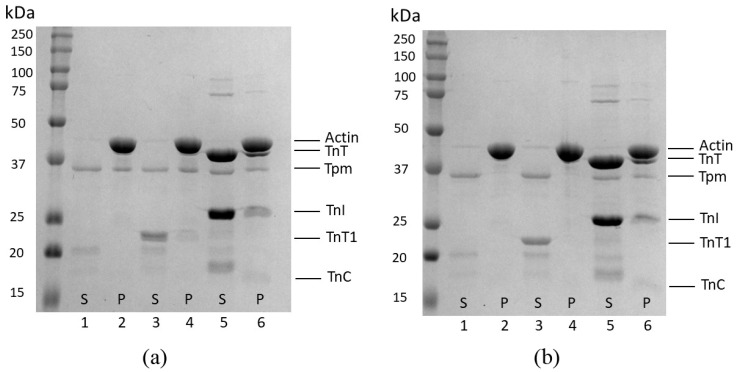
Tpm WT (**a**) and Tpm M8R mutant (**b**) affinities to F-actin in the presence of TnT1 fragment and Tn-complex (with EGTA). Results obtained by co-sedimentation assay. S—supernatants after centrifugation, P—pellets after centrifugation. Lanes 1–2: supernatants (1) and pellets (2) of actin sedimented with Tpm alone; lanes 3–4: supernatants (3) and pellets (4) of actin co-sedimented with Tpm and TnT1; and lanes 5–6: supernatants (5) and pellets (6) of actin co-sedimented with Tpm and whole human cardiac Tn complex.

To study the influence of cardiomyopathy Tpm mutations on the Ca^2+^ regulation of the actin–myosin interaction, we analyzed the Ca^2+^ dependence of the sliding velocity of thin filaments on myosin in the in vitro motility assay (Figure 6 and Table 3). The M8R Tpm mutation decreased the maximum sliding velocity of the filaments (Table 3). In addition, it did not affect pCa_50_ but resulted in incomplete inhibition of the velocity at low Ca^2+^ concentration. The presence of the pCa^2+^-velocity curve for this Tpm mutant in the in vitro motility assay is very surprising because Tpm M8R’s affinity to F-actin was extremely low and the TnT1 fragment was unable to restore it (see Figure 1c,d). Since the thin filament consists of actin, Tn complex, and Tpm, we suggest that the full Tn complex can attach Tpm to the surface of the actin filament. The co-sedimentation of Tpm M8R with F-actin in the presence of Tn showed that Tpm M8R bound to Tn–F-actin filament in the same manner as Tpm WT did (Figure 7a,b, lanes 6), thus confirming our suggestion. 

**Figure 8:**
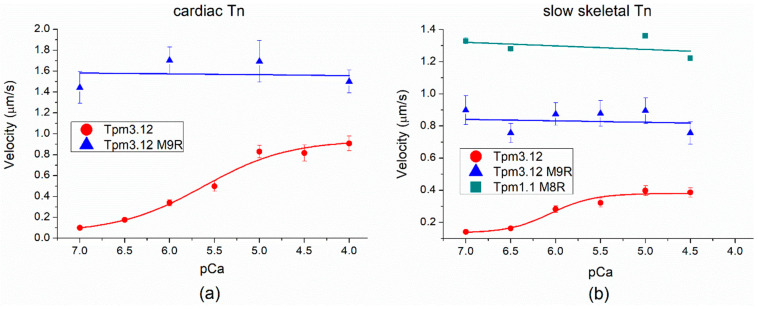
The Ca^2+^-dependent sliding velocity of regulated thin filaments in the presence of cardiomyopathy-causing mutant M8R of Tpm1.1 or myopathy-causing mutant M9R of Tpm 3.12. (**a**) The system contains cardiac Tn complex and cardiac myosin; (**b**) the system contains slow skeletal Tn complex and cardiac myosin. The pCa_50_ values at which the sliding velocity was half-maximal are shown in Table 4.

The Tpm1.1 M8R mutation is a homolog of the Tpm3.12 M9R mutation which causes nemaline myopathy [26,27]. It was shown previously that Tpm3.12 M9R is not able to regulate thin filament movement in the in vitro motility assay [28]. However, our in vitro motility assay data clearly showed that Tpm M8R, in contrast to Tpm3.12 M9R, demonstrates the Ca^2+^ regulation of the actin–myosin interaction (Figure 6b). To clarify this phenomenon, we made an in vitro motility assay by using the following protein compositions: (1) Tpm3.12 M9R with both cardiac Tn and slow skeletal muscle Tn (Figure 8a,b, Table 4) and (2) Tpm1.1 M8R with slow skeletal muscle Tn (Figure 8b and Table 4). The F-actin and cardiac myosin preparations were the same. The Tpm3.12 WT showed Ca^2+^-dependence of the sliding velocity with both cardiac (Figure 8a) and slow skeletal Tn (Figure 8b). However, the sliding velocity of thin filaments with the Tpm3.12 M9R mutation did not depend on the Ca^2+^ concentration with both troponins (Figure 8). The same effect was observed for Tpm1.1 M8R with slow skeletal Tn (Figure 8b).

Two other DCM-causing Tpm mutations, K15N and A277V, lowered the pCa_50_ of the sliding velocity compared to Tpm WT. However, their effect on the maximum filament velocity was different; the K15N mutation increased it by 10% (Figure 6b) and A277V did not affect it (Figure 6a).

HCM mutations I284V and M281T showed incomplete inhibition of the velocity of the filaments at low Ca^2+^. At the same time, they differently influenced the maximum sliding velocity and the Ca^2+^-sensitivity; M281T increased both of them, whereas I284V had no effect (Figure 6a).

### 2.6. Thermodynamics of Tpm Interactions with TnT1

We measured the thermodynamic parameters of TnT1 interaction with Tpm using the isothermal titration calorimetry (ITC) (Table 5 and Table 6). The dissociation constant K_D_ for TnT1 binding to Tpm WT was 0.91 ± 0.14 µM (Table 5). All Tpm mutants bound TnT1 with a K_D_ similar to that of Tpm WT (including Tpm M8R). As TnT1 did not restore the overlap junction of Tpm M8R and its affinity to F-actin (Figure 1 and Table 2), we studied the thermodynamics of the TnT1 interaction with Tpm in detail. Since TnT site 1 binds the Tpm N-to-C overlap junction, we studied two Tpm fragments: N-terminal (Tpm WT_1–133_) and C-terminal (Tpm WT_134–284_). The design of truncated recombinant proteins was determined by trypsin cleavage at Arg133.

TnT1 binding to Tpm caused a viscosity increase. With the use of these truncated proteins, we excluded Tpm polymerization but not the formation of the overlap junction between N- and C-ends, as shown by the K_D_ values of the interaction between Tpm WT_1–133_ and Tpm WT_134–284_ (Table 5). The K_D_ of TnT1 binding to a premixed complex of Tpm WT_1–133_ and Tpm WT_134–284_ was 0.90 ± 0.15 µM (Table 6), which directly matched the K_D_ of TnT1 interaction with the whole Tpm WT (Table 5).

Then we measured the K_D_ of the separate binding of TnT1 to Tpm WT_1–133_ and Tpm WT_134–284_ and found that Tpm WT_1–133_ is unable to bind TnT1, while Tpm WT_134–284_ bound it with a K_D_ of 1.9 ± 0.5 µM. These data can explain the mode of TnT1 binding to Tpm M8R. The ITC data show that Tpm M8R is still able to bind TnT1 at the Tpm C-end; however, it prevents the overlap junction formation and valid attachment of binding site 1. These results are supported by the viscosity measurements (Table 2). We also checked this hypothesis of one-end binding of the M8R Tpm to TnT1 by measuring thermodynamic parameters of Tpm WT_1–133_ or Tpm M8R_1–133_ binding to premixed TnT1 and Tpm_134–284_ complexes. In the case of Tpm M8R_1–133_, we did not observe any binding curve, while Tpm WT_1–133_ interacted with premixed TnT1 and Tpm_134–384_ complex with a K_D_ of 1.7 ± 0.4 µM.

## 3. Discussion

The end-to-end overlap junction is known as an important region for Tpm functioning. Apart from its role in the formation of the Tpm strand and stabilization of the thin filament, it also binds site 1 of TnT. There are multiple mutations in both ends of this junction of the Tpm1.1 molecule, which induce cardiomyopathies. Here, we have investigated the effects of earlier studied Tpm 1.1 cardiomyopathy mutations M8R, K15N, A277V, M281T, and I284V on the Tpm interaction with TnT1.

### 3.1. Mechanism of TnT1 and Tpm Interactions

As known from previous works, the Tpm overlap area binds to site 1 of TnT, and the molecular docking revealed possible interactions between TnT and Tpm amino acid residues [29]. By measuring the K_D_ of direct binding of TnT1 to the N- and C-end of Tpm, we provide the detailed mechanism of interaction between the Tpm strand and TnT site 1. The binding of TnT1 to the Tpm overlap junction stabilizes this area and increases the Tpm viscosity up to eight times (see Table 2) [30,31]. The ITC data revealed the impact of both the N- and C-end on this interaction: the Tpm C-end alone binds TnT1, while the N-end alone does not bind it (see Table 6). The suggestion that the Tpm C-terminal region is critical for Tn binding has been discussed [32,33,34]. The striated muscle Tpm has a unique 9a exon that determines the Tpm interaction with Tn [32]. Replacement of 9a exon by 9d exon (found in smooth muscle Tpm1.3, Tpm1.4, and Tpm1.6–1.8 [26]) results in a decrease in Tpm affinity to actin in the presence of Tn, and most critical for this are the first 18 amino acid residues (258–275) of 9a exon [33,35]. To measure the K_D_ of Tpm interaction with TnT1, we used recombinant Tpm_1–133_ and Tpm_134–284_ proteins. Initially, the approach to separate N- and C-ends into two different peptides was proposed by Palm et al. [24]. Using the first 14 amino acid residues (1a exon) from the N-end and the last 36 amino acids (9a exon) from the C-end of Tpm, they showed that the thermal stability of the mixture of TnT_70–170_ with Tpm C-end is slightly higher than that for the mixture of TnT_70–170_ with Tpm N-end, while the highest stability was found for TnT_70–170_ complex with Tpm overlap. The two possibilities for Tpm N-end to fit in the Tpm-TnT1 structure are: (1) to be part of the whole Tpm overlap junction or (2) to bind the preformed complex of Tpm C-end and TnT1. Our ITC results with Tpm_1–133_ and Tpm_134–284_ fragments support both these ideas. Thus, effective complex formation between the TnT1 fragment and the N- and C-ends of Tpm was observed only in two cases: (1) TnT1 fragment binds the preformed Tpm overlap junction (Tpm WT_1–133_/Tpm WT_134–284_; Table 6) or (2) the N-end of Tpm binds a preformed complex of C-end with TnT1 (TnT1/TpmWT_134–284_; Table 6). From the ITC data, one can conclude that, at first, the Tpm C-end binds TnT1, and only then does the N-end join the Tpm-TnT1 complex. The first N-terminal residues of Tpm and their acetylation are essential for the properties of this ternary complex [34].

### 3.2. Effects of Tpm1.1 M8R Mutation

Significant results were obtained for Tpm M8R mutation. Previously, we have shown that M8R mutation decreased the Tpm affinity to the actin filament and did not affect the sliding velocity of F-actin–Tpm filaments without Tn [20]. Here, we observed that in the presence of TnT1, Tpm M8R does not bind F-actin, unlike all other studied Tpm mutants (Figure 1 and Figure 5). The ITC data pointed out that TnT1 can bind to Tpm M8R (Table 5), but only with its C-end (Table 6), and that leads to the destabilization of the overlap junction because TnT1 cannot connect the ends of the neighboring Tpm dimers. This data is in good agreement with the viscosity measurements (Table 2) and co-sedimentation assay (Figure 1 and Figure 7b, lane 4). Interestingly, these changes did not alter the Ca^2+^-sensitivity of myosin interaction with the thin filament in the in vitro motility assay in our work (Figure 6b; Table 3) and studies of others [18,36]. Racca et al. explained such results by the presence of myosin in the assay and confirmed this suggestion by co-sedimentation of F-actin and Tpm M8R with isolated myosin heads (myosin subfragment 1, S1). Their experiments showed that myosin S1 slightly promoted an interaction between Tpm M8R and F-actin. In the current study, we discovered that not only myosin, but also troponin, influences the interaction between Tpm M8R and F-actin. In the presence of cardiac Tn complex, Tpm M8R interacts with F-actin almost as effectively as Tpm WT (Figure 7a, lane 6 and Figure 7b, lane 6). The whole Tn complex has a full-length TnT and, therefore, two binding sites for F-actin. A Tpm region that interacts with the TnT site 2 is located far from the M8R mutation and can provide the binding of TnT to Tpm with further interaction with the actin filament. In addition, TnI anchors the Tpm-TnT complex on actin.

Destabilization of the overlap junction results in impairment of the activation propagation along the Tpm strand on the actin surface that in turn disrupts Ca^2+^-regulation of the actin–myosin interaction. The Tpm1.1 M8R mutation decreased the maximum sliding velocity of thin filaments in the in vitro motility assay and did not inhibit the velocity at low Ca^2+^ concentrations (pCa from 8 to 6.5, Figure 6b). Such changes in the actin–myosin interaction might be a reason for heart muscle pathology.

### 3.3. Comparison of the Effects of Cardiomyopathy-Causing Tpm1.1 M8R and Myopathy-Causing Tpm3.12 M9R Mutations

The N-terminal sequences of Tpm1.1 (α-Tpm) and Tpm3.12 (γ-Tpm) are virtually identical. Substitutions of Met8 in Tpm1.1 and its respective Met9 in Tpm3.12 of slow skeletal muscles with Arg [26] are associated with two different muscle diseases: cardiomyopathy and nemaline myopathy, correspondingly.

One would expect that at the level of isolated proteins, the functional effects of these two Tpm mutants should be identical. It turned out, however, that that is not the case; their regulatory properties were surprisingly different.

Unlike Tpm1.1 M8R, Tpm3.12 M9R did not have the ability to regulate Ca^2+^ in the sliding velocity of thin filaments [28]. We supposed that such differences might occur due to the properties of Tn complexes. The cardiac, slow skeletal, and fast skeletal muscles have tissue-specific TnT, TnI, and TnC isoforms. To explain such differences in the regulatory properties of Tpm1.1 and Tpm3.12 with these mutations, we have applied the in vitro motility assay with cross-troponin systems [37]: (1) Tpm1.1 M8R with slow skeletal muscle Tn and (2) Tpm3.12 M9R with cardiac muscle Tn.

Tpm3.12 M9R lost its ability to regulate thin filament motility both in the presence of a slow skeletal Tn complex [28] and the cardiac Tn complex (Figure 8a and Table 4). At the same time, Tpm1.1 M8R kept the ability to regulate thin filament motility with cardiac Tn but lost it with slow skeletal Tn (Figure 7b and Table 3).

From these two facts, we assume that the isoform composition of Tpm and Tn plays a key role in the pathogenesis of diseases caused by Tpm1.1 M8R and Tpm3.12 M9R.

In ontogenesis, several Tn isoforms in cardiac tissue are expressed. For example, the slow skeletal TnI isoform expressed in a fetus’s cardiac tissue was replaced by cardiac TnI after birth [38]. The tissue-specific isoforms of TnT interact differently with Tpm. Amarasinghe et al. [39], using sets of truncated TnT proteins, discovered that TnT2 of mouse cardiac and fast and slow skeletal TnT (cTnT, fsTnT, and ssTnT, respectively) bind Tpm similarly. However, TnT1 affinity to Tpm reduced in the following order: fsTnT > cTnT > ssTnT. Cardiac TnT has a 32-amino acid N-terminal extension rich in negative charges and may be significant for the cardiac-specific role of TnT. Thus, we can assume the importance of the N-terminal part of TnT in the interaction with Tpm.

### 3.4. Effects of Tpm1.1 K15N and A277V Mutations

Based on the model suggested by Pavadai et al. [29], the complex of Tpm with TnT is stabilized by electrostatic salt bridges and van der Waal’s and non-polar contacts. In this model, the Tpm Glu16 amino acid residue forms an electrostatic contact with Arg84 of TnT. The head-to-tail overlap junction of α-Tpm mainly recruits the first eight amino acid residues of one Tpm dimer and the last seven–eight residues of another. Based on this model, Lys15 is not located directly in the overlap junction, but it is required for forming a bifurcation and, therefore, for the Tpm polymerization. This was approved by studies indicating the low viscosity of Tpm K15N and so its decreased affinity to F-actin [19,20].

Here, we have shown that K15N substitution significantly affects the ability of Tpm to interact with TnT, particularly with site 1. However, even in the presence of TnT1, Tpm K15N did not restore the viscosity, and the K_50%_ of its binding to F-actin was retained higher than that with Tpm WT. On the other hand, the TnT1 interaction with Tpm K15N substantially stabilized the complex of this Tpm mutant with F-actin (Figure 4 and Table 1). These effects could result in an impaired actin–myosin interaction, seen by a lower Ca^2+^-sensitivity in the in vitro motility assay compared with Tpm WT, both in our experiments and other studies [23].

Similar effects were observed with Tpm A277V in the in vitro motility assay (Figure 6a); however, in all other types of experiments, the effects of Tpm A277V and Tpm K15N were different (Table 1 and Figure 2). The decrease in Ca^2+^-sensitivity of the myosin–actin interaction with Tpm K15N and A277V fit the dilated cardiomyopathy phenotype found in patients with DCM. 

The effects of Tpm A277V can relate to the stiffness of the Tpm strand, which in turn correlate with Tpm viscosity. Formation of the overlap junction leads to polymerization of the Tpm strand; the higher viscosity, the longer and stiffer the Tpm strand. The viscosity of Tpm A277V without TnT1 was higher than that of the Tpm WT, resulting in Tpm A277V more strongly suppressing the motility of F-actin filaments in the in vitro motility assay compared to Tpm WT (Figure 5). However, the addition of TnT1 decreased the velocity of the filaments with Tpm WT to a greater extent than with Tpm A277V (Figure 5), as in the presence of TnT1, the viscosity of Tpm WT was higher than that of Tpm A277V (Figure 4 and Table 2).

### 3.5. Effects of Tpm1.1 M281T and I284V Mutations

The Tpm1.1 M281T and I284V mutations are known to cause the HCM phenotype, which is characterized by an increased Ca^2+^-sensitivity of the actin–myosin interaction (Table 3). Both these mutations increased the pCa_50_ of the sliding velocity of thin filaments and did not inhibit the velocity at low Ca^2+^ concentrations (at pCa from 8 to 6.5; Figure 6a; Table 3). The higher Ca^2+^ sensitivity of the thin filament with Tpm M281T and I284V was also observed previously [40,41]. The functional significance of the N-terminal part of TnT on the activation of the thin filament and kinetics of cross-bridge attachment was shown previously [16]. It was demonstrated that the TnT1 fragment decreases the ATPase activity of myosin and the sliding velocity of Tpm-F-actin filaments in the in vitro motility assay [14,15]. M281T and I284V mutations are located in the zone of interaction between Tpm and TnT1. To study the effect of TnT1 on the actin–myosin interaction, we have analyzed the dependence of the sliding velocity of F-actin-Tpm filaments on the TnT1 concentration in the in vitro motility assay (Figure 5). We found that TnT1 decreased the velocity of the filaments with Tpm A277V and Tpm M281T to a lesser degree than with Tpm WT and did not affect the velocity of filaments containing the Tpm with M8R, K15N, and I284V mutations. The ITC data showed that TnT1 equally interacts with Tpm WT, M281T, and I284V. Actin and myosin contribute to the TnT1 interaction with Tpm. In addition, Tpm, as it was shown, directly interacts with myosin in each quasi-repeat, and mutations affect this interaction [42]. Therefore, the effects of M281T and I284V Tpm mutants can be accounted for by an alteration in myosin interaction with the thin filament. In addition, the effects on the velocity in the motility assay can be due to the difference in the TnT1 influence on the stiffness of the Tpm strand with these mutations. The estimated viscosity of Tpm M281T and I284V with TnT1 was lower than that of Tpm WT (Figure 4 and Table 2), which correlates with the suppression of motility with TnT1.

## 4. Materials and Methods

### 4.1. Protein Preparations

All Tpm constructs were obtained in the pMW 172 vector. Human Tpm1.1 (α-Tpm) constructs, containing cardiomyopathy-causing mutations were obtained as described in [20]. All coding sequences were cloned between the NdeI and EcoRI restriction sites. The coding sequences of Tpm were preceded by the 5′-GCTAGC-3′ corresponding to the Ala-Ser dipeptide, imitating the naturally occurring N-terminal acetylation, which is necessary for the interaction of Tpm with actin [43,44]. Tropomyosin N-terminal (Tpm_1–133_) and C-terminal (Tpm_134–284_) fragments were obtained from the coding sequence of full Tpm1.1 by using the following primers: 5′-GCCCAAAAAGATGAAGAAAAAATGG-3′ as forward and 5′-CATATGTATATCTCCTTCTTAAAGTTAAACAAAATTA-3′ as reverse primers for Tpm_134–284_ and 5′-TAAGAATTCCGATCCGGCTGC-3′ as forward and 5′-TCGACTCTCAATGACTTTCATGCC-3′ as reverse primers for Tpm_1–133_.

Human adult cardiac troponin T (TNT3, or isoform 6 of P45379) fragment TnT1 (or TnT1–158) was obtained from the coding sequence of full human cardiac TnT by using following primers: 5′-ATATATCATATGCATCACCATCACCATCACCTGGAAGTGCTGTTTCAGGGCCCGATGAGCGACATTGAAGAAGTGGTGGAAG-3′ as forward and ATATATGAATTCTCAACGGCGCGCACGTTCTTC as reverse primers. The coding sequence of the TnT fragment contained His-tag and a cleavage site for 3C protease. The PCR product was cloned between the NdeI and EcoRI restriction sites in the pET23a+ plasmid. All Tpm and TnT fragment constructs were sequenced.

Recombinant Tpm proteins were expressed in *E. coli* C41 (DE3) bacterial cells and purified by anion exchange chromatography using a HiTrap Q HP column as described earlier [45]. Tpm concentration was determined spectrophotometrically at 280 nm using the following extinction coefficients: E^1%^ of 2.73 cm^−1^ for Tpm1.1 WT and mutants, E^1%^ of 0.98 cm^−1^ for Tpm_1–133_, and E^1%^ of 4.23 cm^−1^ for Tpm_134–184_. According to SDS-PAGE, the purity of all the Tpm preparations was no less than 95%. The purified proteins were stored at −80 °C. The Tpm species were reduced before all experiments by heating with 4 mM DTT at 60 °C for 15 min.

His-TnT1 was expressed in *E. coli* C41 (DE3) bacterial cells and purified by using a HisTrap column and an imidazole gradient from 15 to 500 mM. The His-tag was removed by incubation with 3C-protease in the weight ratio 3C-protease: His-TnT1 of 1:400 at 4 °C for 4 h. The purified TnT1 protein was dialyzed against 30 mM HEPES-Na buffer (pH 7.3) containing 100 mM NaCl and stored at −80 °C. The purity of the obtained protein was no less than 95%. TnT1 concentration was determined spectrophotometrically at 280 nm using an E^1%^ of 0.71 cm^−1^. 

Recombinant human cardiac troponin TIC-complex (cTn) was provided by HyTest (Cat.# 8ITCR). The concentration of the Tn complex was determined by spectrophotometry at A_280_ using an extinction coefficient of 0.99 for cTn complex containing 1 mg/ml cTnI.

### 4.2. Co-Sedimentation and Quantitative Electrophoresis

The affinity of Tpm species to F-actin in the presence of TnT1 was estimated by a co-sedimentation assay as previously described [20]. The F-actin in a 10 μM concentration was mixed with TnT1 to the final concentration of 1.5 μM and Tpm in concentration increasing from 0 to 7.5 μM at 20 °C in 30 mM HEPES-Na buffer (pH 7.3) containing 255 mM NaCl (for Tpm WT, A277V, M281T, and I284V mutants) or 200 mM NaCl (for Tpm with M8R and K15N mutations) to a final volume of 100 μL. Tpm concentration is given for dimer. After 40 min incubation, actin was pelleted with any bound Tpm by ultracentrifugation at 100,000× *g* for 40 min. Equivalent samples of the pellet and the supernatant were subjected to SDS-PAGE. Protein bands were scanned and analyzed using ImageJ 1.53k software (Scion, Frederick, MD, USA).

### 4.3. Light Scattering

The stability of Tpm–F-actin complexes in the presence of the TnT1 fragment was measured by thermally induced changes in the light scattering. The measurements were performed at 350 nm and a constant heating rate of 1 °C/min on a Cary Eclipse fluorescence spectrophotometer (Varian Australia Pty Ltd., Mulgrave, Australia) equipped with a temperature controller and thermoprobes. The solutions contained the same concentration of F-actin (20 μM) and Tpm (10.5 μM) species in the absence or the presence of TnT1 (60 μM) in 30 mM HEPES-Na buffer (pH 7.3) containing 100 mM NaCl. During the heating, Tpm and the TnT1 fragment dissociate from F-actin and the value of the light scattering intensity became equal to that of F-actin alone. The temperature dependence of the light scattering of F-actin alone was subtracted from experimental curves. The resulting curves were analyzed by fitting them to a sigmoidal decay function (Boltzman) using Origin 7.0 software. The main parameter which was taken into analysis was T_diss_, i.e., the temperature at which a 50% decrease in light scattering occurs.

### 4.4. Viscosity Measurements 

The viscosity measurements were performed on a falling ball microviscometer Anton Paar AMVn (Ashland, VA, USA) in 0.5 mL capillary at 20 °C. The specific density of the Tpm solutions was measured with an Anton Paar DMA 4500 device (Ashland, VA, USA) and taken into account for an accurate viscosity calculation. All measurements were performed at a Tpm concentration of 1.0 mg/mL (15 µM) in 30 mM HEPES-Na buffer (pH 7.3) containing 100 mM NaCl and 4 mM DTT. The molar ratio Tpm/TnT1 was 1/1. The measurements for each sample were repeated three times, and the obtained values were averaged.

### 4.5. Isothermal Titration Calorimetry (ITC)

The thermodynamic parameters of TnT1 binding to Tpm fragments and mutants were measured using a MicroCal PEAQ-ITC instrument (Malvern Panalytical, Worcestershire, UK), as described elsewhere [46]. Experiments were carried out at 25 °C in 30 mM HEPES-Na buffer (pH 7.3) containing 100 mM NaCl and 1 mM DTT. The protein concentration in the cell (marked as a sample) ranged from 30 to 60 µM and the protein concentration in the syringe (marked as a ligand) ranged from 200 to 700 µM. The samples and ligands for each experiment are described in Table 5 and Table 6. Ligands of 2.5 μL aliquots were injected from a 40-μL syringe into a 0.2 mL cell containing protein solution to achieve a complete binding isotherm. The heat of dilution was measured by injection of the ligand into the buffer solution. The resulting titration curves were fitted using MicroCal PEAQ-ITC Analysis Software, assuming one set of binding sites. Binding constants (K_D_), stoichiometry (N), and enthalpy variations (ΔH) were determined by a non-linear regression fitting procedure. The Gibbs energy (ΔG) and entropy variations (ΔS) were calculated from the equation: RTlnK_D_ = ΔG = ΔH − TΔS.

### 4.6. In Vitro Motility Assay

Actin from m. psoas of a rabbit, myosin from the left ventricle of a lamb, and the Tn complex from a pig’s left ventricle or m. soleus of a rabbit were prepared by standard methods [47,48]. F-actin was stabilized by the addition of 1.5-fold molar excess of phalloidin. For experiments in the in vitro motility assay, F-actin was labeled with phalloidin-tetramethylrhodamine B isothiocyanate in a 1:2 molar ratio (Sigma Chemical Co., St Louis, MO, USA).

The in vitro motility assay was performed as described previously [28]. The myosin (300 µg/mL) in AB buffer (25 mM KCl, 25 mM imidazole, 4 mM MgCl_2_, 1 mM EGTA, and 20 mM DTT, pH 7.5) containing 0.5 M KCl was loaded into an experimental flow cell with the nitrocellulose-coated inner surface. After 2 min, 0.5 mg/mL BSA was added for 1 min. Non-labeled F-actin in AB buffer with 2 mM ATP was added for 5 min. To form regulated thin filaments, 10 nM phalloidin-tetramethylrhodamine B isothiocyanate-labeled F-actin and 100 nM Tpm/Tn were added for 5 min. Unbound filaments were washed out with AB buffer. Finally, the cell was washed with AB buffer containing 0.5 mg/mL BSA, oxygen scavenger system (3.5 mg/mL glucose, 20 μg/mL catalase, and 0.15 mg/mL glucose oxidase), 20 mM DTT, 2 mM ATP, 0.5% methylcellulose, 100 nM Tpm/Tn, and appropriate Ca^2+^/EGTA in proportions calculated with Maxchelator (http://www.stanford.edu/~cpatton/webmaxc/webmaxcS.html, accessed on 1 November 2021) program.

The interaction of TnT1 with the F-actin–Tpm filaments was studied by analyzing the dependence of the filament velocity on the concentration of TnT1. The final AB buffer contained 2 mM ATP, 5 nM phalloidin-tetramethylrhodamine B isothiocyanate-labeled F-actin, 100 nM Tpm, and TnT1 in the concentrations of 0, 50, and 100 nM. 

The experiments were carried out at 30 °C. For each flow cell, ten 30 s image sequences were recorded from different fields containing ~30–50 thin filaments. At saturating Ca^2+^ concentrations, all filaments in each field of view were moving smoothly, but with a decreased Ca^2+^ concentration, the amount of uniformly moving filaments gradually decreased. The filaments sliding velocities were measured using the GMimPro software. The means of individual experiments were fitted to the Hill equation: *V* = *V*_max_ × (1 + 10^n(pCa-pCa50)^)^−1^, where V and V _max_ are the velocity and the maximal velocity at saturating calcium concentration, respectively; pCa_50_ (i.e., calcium sensitivity) is pCa at which half-maximal velocity is achieved; and n is the Hill coefficient. The parameters of individual experiments were averaged.

## 5. Conclusions

The mutations located in the C- and N-ends of the Tpm overlap junction cause dilated and hypertrophic cardiomyopathies. The mechanisms by which these substitutions result in pathology may differ. Here, we investigated the role of TnT in the development of cardiomyopathies caused by the Tpm mutations in the overlap junction. We have demonstrated the influence of TnT on the functional manifestations of Tpm M8R, K15N, and A277V mutations. In the case of the M8R mutation, we demonstrated the key role of Tn in its pathogenic effects. We found that the isoform composition of the Tn complex plays an essential role in cardiomyopathy development.

For pathogenic mutants Tpm M281T and I284V we did not find any significant changes in the interaction with TnT1. However, the most pronounced effects of these Tpm mutants were observed in the in vitro motility assay, which also contains myosin as well as F-actin, Tn, and Tpm. Therefore, the pathogenic effects of Tpm M281T and I284V might be related with the alterations in the interaction of the myosin head with F-actin.

## Figures and Tables

**Table 1:** Thermal stability of Tpm–F-actin complexes in the presence or absence of TnT1 fragment.

Tpm	T_diss_, °C without TnT1	T_diss_, °C with TnT1
WT	46.5 ± 0.03	48.1 ± 0.02
M8R	ND	ND
K15N	39.4 ± 0.06	44.1 ± 0.04
A277V	48.7 ± 0.06	50.3 ± 0.04
M281T	45.4 ± 0.03	46.8 ± 0.02
I284V	45.4 ± 0.03	47.7 ± 0.04

**Table 2:** Excess viscosities of Tpm mutants’ solutions in the presence or absence of TnT1 over buffer viscosity.

Tpm	Viscosity (mPa·s) without TnT1	Viscosity (mPa·s) with TnT1
WT	0.125 ± 0.001	0.992 ± 0.001 ^#^
M8R	0.018 ± 0.001 ^§^	0.033 ± 0.001 ^§,#^
K15N	0.043 ± 0.001 ^§^	0.295 ± 0.007 ^§,#^
A277V	0.195 ± 0.001 ^§^	0.783 ± 0.023 ^§,#^
M281T	0.064 ± 0.001 ^§^	0.439 ± 0.002 ^§,#^
I284V	0.102 ± 0.001 ^§^	0.830 ± 0.003 ^§,#^

^§^ Significant differences in the excess viscosity of Tpm mutant from that of Tpm WT, *p* < 0.05; ^#^ Significant differences in the excess viscosity of Tpm with TnT1 from that of Tpm alone, *p* < 0.05.

**Table 3:** Effects of Tpm mutations on the characteristics of the pCa-velocity dependence.

Tpm	V_max_ (µm/s)	V_0_ (µm/s)	h	pCa_50_
WT	3.7 ± 0.1	0	2 ± 0.2	6.33 ± 0.01
M8R	2.8 ± 0.1 *	1.0 ± 0.1 *	2.2 ± 0.2	6.33 ± 0.03
K15N	4.0 ± 0.1 *	0.2 ± 0.1	1.8 ± 0.3	5.85 ± 0.01 *
A277V	3.7 ± 0.1	0.2 ± 0.1	1.6 ± 0.2	5.68 ± 0.01 *
M281T	4.6 ± 0.1 *	0.8 ± 0.1 *	2.7 ± 0.3	6.47 ± 0.01 *
I284V	3.7 ± 0.1	1.0 ± 0.1 *	2.1 ± 0.2	6.34 ± 0.01

* V_max_, the maximal sliding velocity of thin filaments at saturating Ca^2+^ concentration; V_0_, sliding velocity at low Ca^2+^ concentrations; pCa_50_, pCa at which V is half-maximal; h, coefficient of cooperativity. Parameters of the Hill equation are represented as mean ± S.D. The * symbol indicates the significant difference in the parameters measured with the Tpm with mutations from those with WT Tpm, *p* < 0.05.

**Table 4:** Effects of Tpm mutations on the characteristics of the pCa-velocity dependence in the presence of cardiac or slow skeletal Tn complex.

Myosin and Tn	Tpm	V_max_ (µm/s)	pCa_50_
cardiac myosin + cardiac Tn	γ-Tpm	0.94 ± 0.08	5.62 ± 0.12
	M9R γ-Tpm	1.55 ± 0.17	
cardiac myosin + slow skeletal Tn	γ-Tpm	0.38 ± 0.02	6.05 ± 0.11
	M9R γ-Tpm	0.84 ± 0.06	
	M8R α-Tpm	1.30 ± 0.06	

V_max_, the maximal sliding velocity of thin filaments at saturating Ca^2+^ concentration; pCa_50_, pCa at which V is half-maximal.

**Table 5:** Thermodynamic parameters of the interaction of TnT1 (as a ligand) with Tpm mutants at 25 °C determined by ITC.

Sample	N	K_D_, µM	ΔH, kcal/mol	ΔG, kcal/mol	TΔS, kcal/mol
Tpm WT	0.629 ± 0.009	0.91 ± 0.14	−9.69 ± 0.26	−8.25	−1.5
Tpm M8R	1.15 ± 0.03	1.9 ± 0.4	2.51 ± 0.13	−7.8	10.3
Tpm K15N	1.05 ± 0.02	1.3 ± 0.3	−12.1 ± 0.5	−8.0	−4.1
Tpm A277V	1.03 ± 0.01	2.51 ± 0.15	−10.60 ± 0.02	−7.6	−3.0
Tpm M281T	0.857 ± 0.014	1.52 ± 0.21	−8.83 ± 0.28	−7.9	−0.9
Tpm I284V	0.775 ± 0.008	1.89 ± 0.16	−14.40 ± 0.29	−7.9	−6.6

**Table 6:** Thermodynamic parameters of TnT1 and truncated Tpm interaction at 25 °C determined by ITC.

Sample	Ligand	N	K_D_, µM	ΔH, kcal/mol	ΔG, kcal/mol	TΔS, kcal/mol
Tpm WT_1–133_/ Tpm_134–284_	TnT1	0.69 ± 0.012	0.90 ± 0.15	−7.7 ± 0.3	−8.25	0.6
Tpm WT_1–133_	TnT1	no binding	-	-	-	-
Tpm WT_134–284_	TnT1	1.06 ± 0.03	1.9 ± 0.5	2.10 ± 0.11	−7.8	9.9
Tpm WT_134–284_	Tpm WT_1–133_	1.17 ± 0.05	10.2 ± 0.3	1.95 ± 0.15	−6.8	8.8
TnT1/ TpmWT_134–284_	Tpm WT_1–133_	1.07 ± 0.03	1.7 ± 0.4	−6.5 ± 0.4	−7.9	1.4
TnT1/ TpmWT_134–284_	Tpm M8R_1–133_	no binding	-	-	-	-
